# Analysis of the clinical significance of DNA methylation in gastric cancer based on a genome-wide high-resolution array

**DOI:** 10.1186/s13148-019-0747-5

**Published:** 2019-11-01

**Authors:** Wen-Liang Fang, Ming-Huang Chen, Kuo-Hung Huang, Shih-Ching Chang, Chien-Hsing Lin, Yee Chao, Su-Shun Lo, Anna Fen-Yau Li, Chew-Wun Wu, Yi-Ming Shyr

**Affiliations:** 1 Division of General Surgery, Department of Surgery, Taipei Veterans General Hospital, No. 201, Sec. 2, Shipai Rd, Beitou District, Taipei City, Taiwan 11217; 2 School of Medicine, National Yang-Ming University, Taipei City, Taiwan 11217; 3 Department of Oncology, Taipei Veterans General Hospital, Taipei City, Taiwan 11217; 4 Division of Colon & Rectal Surgery, Department of Surgery, Taipei Veterans General Hospital, Taipei City, Taiwan 11217; 5 Genome Research Center, National Yang-Ming University, Taipei City, Taiwan 11217; 6 National Yang-Ming University Hospital, Yilan County, Taiwan 26058; 7 Department of Pathology, Taipei Veterans General Hospital, Taipei City, 11217 Taiwan

**Keywords:** Methylation, Plasma, Recurrence pattern, Distant metastasis, Survival

## Abstract

**Background:**

Aberrant DNA methylation is involved in gastric carcinogenesis and may serve as a useful biomarker in the diagnosis and detection of gastric cancer (GC) recurrence.

**Results:**

A total of 157 patients who received surgery for GC were enrolled in the present study. A genome-wide methylation analysis was performed in tumor and adjacent normal tissues for the discovery set of 16 GC patients; the top three hypermethylated CpG sites of DNA promoters were selected for validation in tissue and plasma samples for the validation set of 141 GC patients. The frequencies of the top three hypermethylated genes in available patient tissues (*n* = 141) and plasma samples (*n* = 106) were 41.8% and 38.7%, respectively, for *ADAM19*; 40.4% and 42.5%, respectively, for *FLI1*; and 56.7% and 50.9%, respectively, for *MSC*. In both tissue and plasma samples, *FLI1* hypermethylation was associated with more advanced GC and liver and distant lymphatic metastasis, and *ADAM19* hypermethylation was associated with more stage IV GC. In plasma samples, *MSC* hypermethylation was more common in non-superficial type GC than samples without *MSC* hypermethylation. In both tissue and plasma samples, patients with methylation of all the three genes had significantly more liver metastases, distant lymphatic metastases, and paraaortic lymph node metastases than patients with two or fewer hypermethylated genes. The survival analysis showed that only for stage III GC, patients with hypermethylation of two or three genes had a worse 5-year disease-free survival rate than those with hypermethylation of one or none of the three genes. Subgroup analysis showed that *FLI1* hypermethylation in both tissue and plasma samples was associated with liver metastasis in MSI−/EBV− GC, and *MSC* hypermethylation in tissue samples was correlated with liver metastasis in MSI+ or EBV+ GC. Patients with *FLI1* hypermethylation in plasma samples had a significantly worse 5-year disease-free survival rate than those without *FLI1* hypermethylation in MSI−/EBV− GC. *FLI1* hypermethylation was an independent prognostic factor affecting the overall survival and disease-free survival in both tissue and plasma samples.

**Conclusions:**

DNA methylation is a useful biomarker for predicting tumor recurrence patterns and GC patient survival.

## Background

Although the worldwide incidence of gastric cancer (GC) has been declining, it remains the sixth most common cancer and the second most common cause of cancer-related death [1]. Despite advances in diagnostic methods and treatment modalities, the prognosis of GC remains unsatisfactory because most patients are diagnosed at an advanced stage. To improve patient survival, useful biomarkers are needed to support the early diagnosis of GC and the early detection of cancer recurrence.

Even in early stage GC, aberrant DNA methylation may contribute to gastric carcinogenesis by silencing of tumor suppressor genes or stimulating the expression of oncogenes [2–4]. A meta-analysis found that several hypermethylated genes were differentially expressed between normal and GC tissues [5]. In addition to their usefulness in tumor tissues, some hypermethylated genes identified in plasma have been correlated with clinical significance in hepatocellular carcinoma (*HOXA1*, *EMX1*, *AK055957*, *ECE1*, *PFKP*, and *CLEC11A*), colorectal cancer (*AGBL4*, *FLI1*, and *TWIST1*), and GC (*ELMO1*, *ZNF569*, and *C13orf18*) [6–8]. However, to date, genome-wide analyses aimed at identifying and validating novel hypermethylated genes in both tissue and plasma samples obtained from GC patients are lacking.

The Cancer Genome Atlas (TCGA) [9] classifies GC into four types: (1) Epstein-Barr virus positive (EBV+), (2) microsatellite instability-high (MSI+), (3) genomically stable, and (4) chromosomal instability. Most of the prognostic DNA methylation markers are hypermethylated in MSI+ and EBV+ GC, and the methylation status of these markers is likely to be associated with good prognosis [9–11]. Although there are several reports of DNA methylation markers associated with poor prognosis [12–16], they are rarely used in clinical practice. Most studies reporting these markers did not demonstrate validation analyses with independent samples and were not supported by subsequent studies by other investigators.

The aim of this study was to use genome-wide methylation analysis to identify novel hypermethylated genes that can discriminate tumors from normal tissues in GC patients. We further validated the top three hypermethylated genes in both tissue and plasma samples and investigated the correlations between hypermethylated genes and the clinicopathological characteristics and recurrence patterns of GC patients. Furthermore, the present study classified GC into subtypes according to MSI/EBV status. The methylation frequencies of the top three hypermethylated genes and their correlation with the clinicopathological characteristics and prognosis among the GC subtypes were compared.

## Results

### Clinicopathological characteristics

**Fig. 1:**
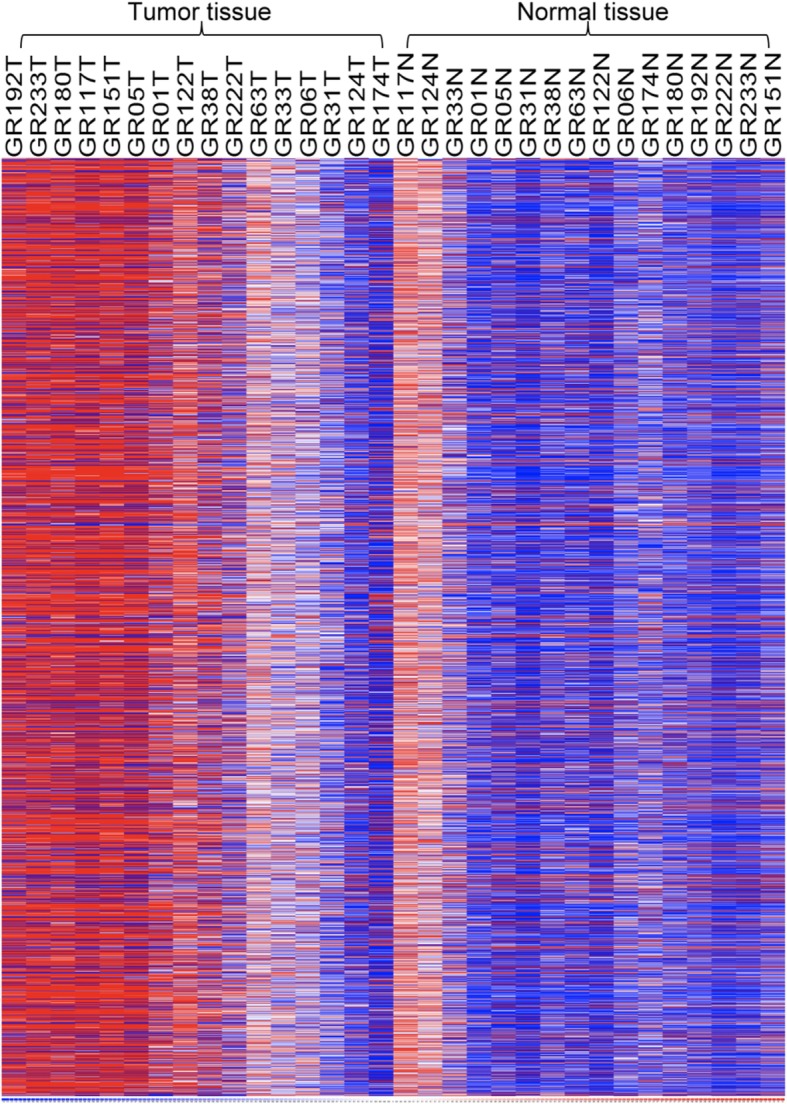
Heatmap of differently methylated CpG sites of tumor and normal tissue of GC patients. Tumors and adjacent non-tumor tissues of 16 GCs, including four patients in each TNM stage, were epigenotyped and analyzed using the Illumina methylation EPIC BeadChip assay. The red color represents the hypermethylated genes, while the blue color indicates the hypomethylated genes. Based on 2180 CpG sites, the methylation patterns of most tumor tissues were hypermethylated, while hypomethylation patterns were observed in most normal tissues. The heatmap was established by dCHIP (https://www.softpedia.com/get/Science-CAD/dChip.shtml)

As shown in Fig. 1, genome-wide Illumina methylation EPIC BeadChip profiling was performed on tumor and adjacent normal DNA samples of the discovery set of 16 GC patients (including four patients in each TNM stage). We compared the methylation patterns between tumor and normal tissues and found that 2180 of 865,918 CpG sites (0.25%) were differentially methylated in the tumor tissues. These CpG sites were within or near 837 functional genes; 78.4% (1710 CpG sites) were hypermethylated, and 21.6% (470 CpG sites) were hypomethylated in the tumor group. EpiTect Control DNA (Qiagen) was used to assess the performance of the MassARRAY EpiTYPING method and primer design. The values of the methylation sites were 0.95 ± 0.09 and 0.02 ± 0.13 in the methylated and unmethylated controls, respectively, indicating that the primer designs were acceptable for this study. Additional file 1 showed the raw data of the results of genome-wide methylation profiling using an Illumina Methylation EPIC BeadChip assay. The data of the hypermethylated and hypomethylated genes that were significantly differentially expressed between the normal and cancer tissues are shown in Additional file 2.

To replicate the results from our genome-wide methylation analysis, we selected the top three hypermethylated CpG islands from the discovery set of 16 GC patients, including A Disintegrin and Metalloproteinase 19 (*ADAM19*, hg19 chr5:157,002,422-157,002,626), Friend Leukemia Integration 1 (*FLI1*, hg19 chr11:128,564,720-128,564,959), and Musculin (*MSC*, hg19 chr8:72,756,005-72,756,149) genes. Validation of the same CpG sites of the three genes was performed in 141 independent GC patients using a MALDI-TOF-based methylation profiling method. The *ADAM19*, *FLI1*, and *MSC* genes were hypermethylated in tumor tissues in 59 (41.8%), 57 (40.4%), and 80 cases (56.7%), respectively. The raw data of the clinical profile of the discovery set (*n* = 16) and validation set (*n* = 141) of GC patients was shown in Additional file 3 and Additional file 4. The clinical profile of the discovery set and validation set was shown in Additional file 5: Table S1. As shown in Additional file 4, the most frequently differentially hypomethylated CpG islands different between the normal and tumor tissues were *TACSTD2*, followed by *SIM2* and *DAPK1*.

#### Tissue samples

**Fig. 2:**
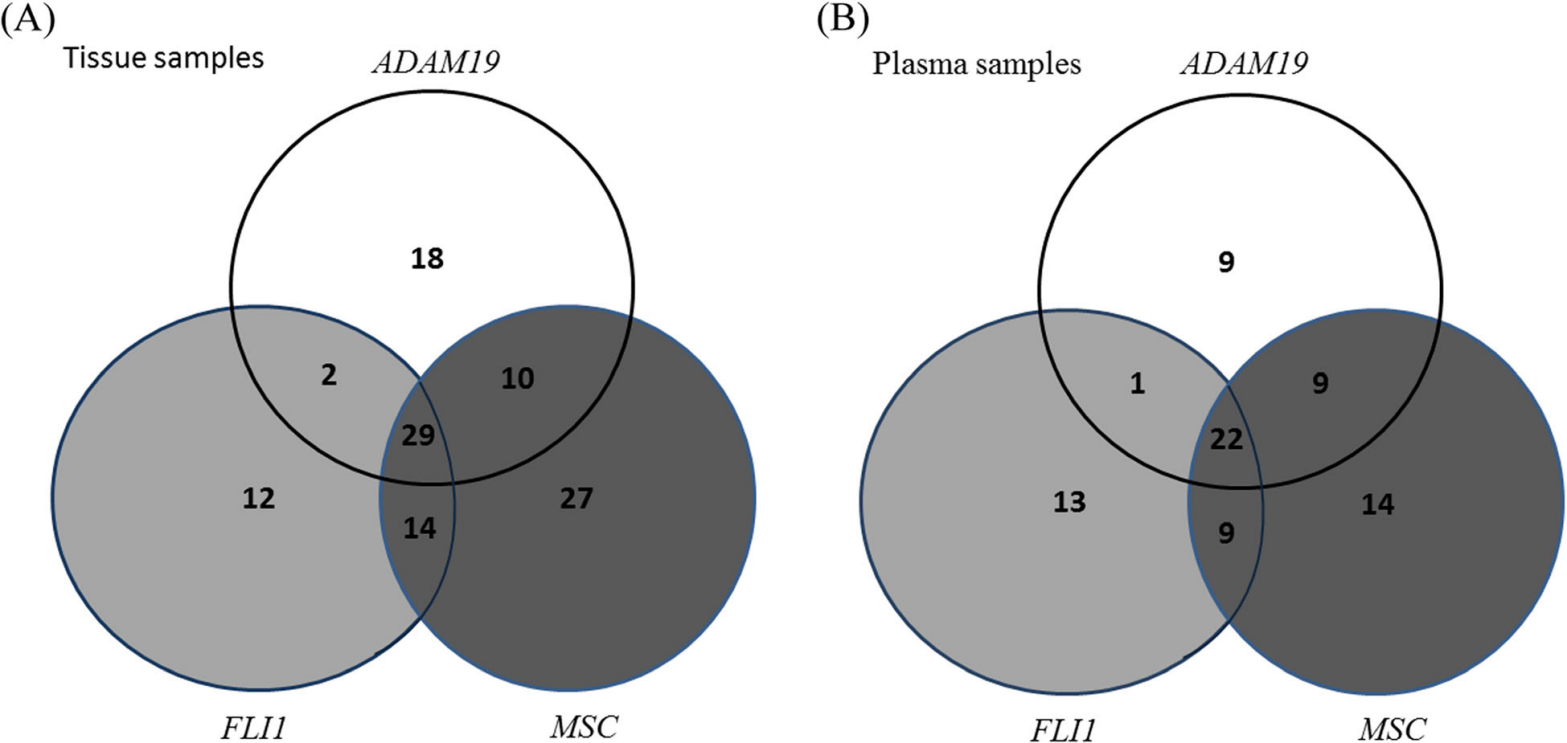
The correlations of the hypermethylation status of the three genes. **a** Tissue samples. **b** Plasma samples

**Fig. 3:**
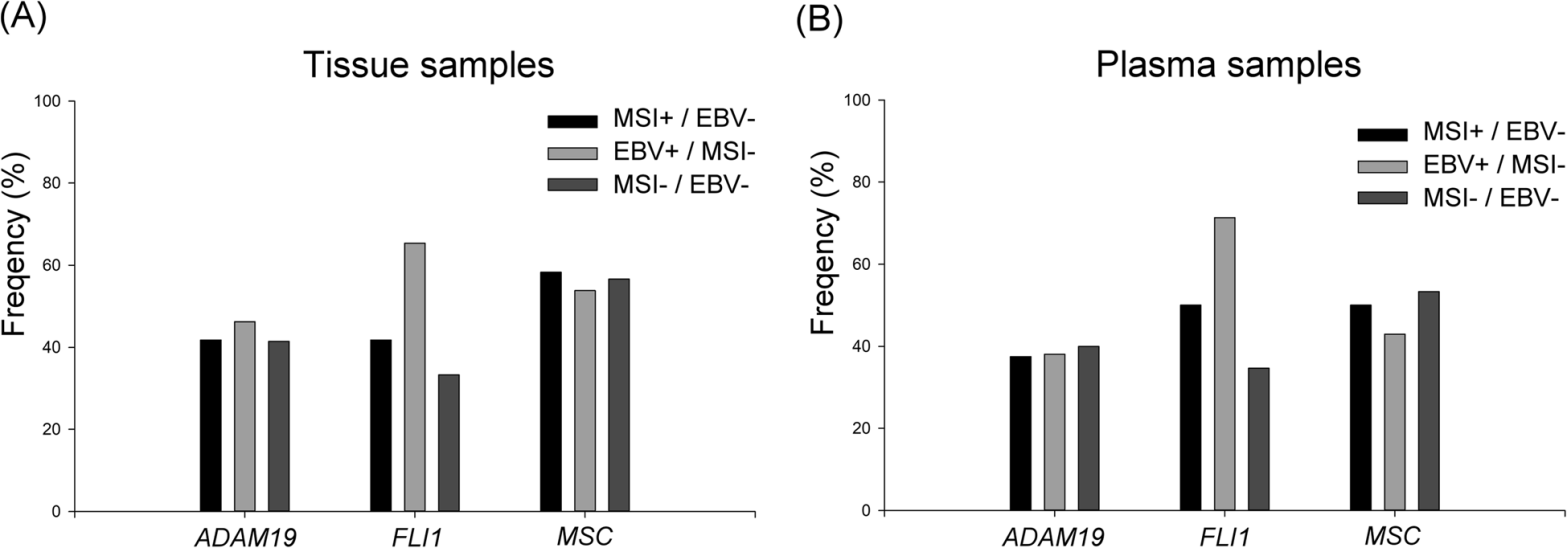
The bar chart of the frequencies of the three methylated genes in different GC subtypes according to MSI/EBV status. **a** Tissue samples. **b** Plasma samples

As shown in Table 1, patients with *ADAM19* hypermethylation more often had stage IV GC than those without *ADAM19* hypermethylation (*P* = 0.043); patients with *FLI1* hypermethylation had fewer early GC (pT1) than those without *FLI1* hypermethylation (*P* = 0.003). No correlations were found between the clinicopathological characteristics and *MSC* hypermethylation in GC patient tissues. The correlations of the hypermethylation status of the three genes in tissue samples were shown in Additional file 6: Table S2 and Fig. 3a. Patients with *ADAM19* hypermethylation were significantly associated with *FLI1* hypermethylation. Patients with *FLI1* hypermethylation were significantly associated with *MSC* hypermethylation (Additional file 6: Table S2). Thirty-one patients had co-hypermethylation of *ADAM19* and *FLI1*; 39 patients had co-hypermethylation of *ADAM19* and MSC; 43 patients had co-hypermethylation of *FLI1* and *MSC*; and 29 patients had co-hypermethylation of the three genes (Fig. 2a).

**Table 1:** Clinical profile in the tissue and plasma samples of gastric cancer patients with hypermethylation of the *ADAM19*, *FLI1*, and *MSC* genes

Variables	Tissue samples							Plasma samples						
	Case no., *n* = 141	*ADAM19* *n* = 59	*P* value	*FLI1* *n* = 57	*P* value	*MSC* *n* = 80	*P* value	Case no. *n* = 106	*ADAM19* *n* = 41	*P* value	*FLI1* *n* = 45	*P* value	*MSC* *n* = 54	*P* value
Age (years)			0.597		0.860		0.860			0.537		0.860		0.686
< 65/≧ 65	51/90	23/36		20/37		28/52		38/68	13/28		15/30		18/36	
Gender			0.163		0.319		0.167			1.000		0.110		0.650
Male/female	107/34	41/18		46/11		57/23		81/25	31/10		38/7		40/14	
Tumor size (cm)			0.247		0.847		0.848			0.827		0.830		0.832
< 5/≧ 5	36/105	12/47		14/43		21/59		31/75	11/30		14/31		15/39	
Cell differentiation			0.814		0.666		0.221			0.843		0.434		0.119
Poor/moderate/well	71/69/1	30/29/0		27/30/0		37/42/1		50/56/0	20/21/0		19/26/0		21/33/0	
Gross appearance			0.069		0.218		0.170			0.154		0.296		*0.025*
Superficial type	18	3 (17.6)		4 (22.2)		7 (38.9)		12	0		3 (25.0)		2 (16.7)	
Borrmann type 1 and 2	30	15 (51.7)		14 (46.7)		20 (66.7)		25	14 (56.0)		13 (52.0)		16 (64.0)	
Borrmann type 3 and 4	93	34 (37.0)		39 (41.9)		53 (57.0)		69	27 (39.1)		29 (42.0)		36 (52.2)	
Lauren’s classification			0.718		1.000		0.591			0.667		1.000		0.591
Intestinal/diffuse type	94/47	38/21		38/19		55/25		75/31	28/13		32/13		55/25	
Lymphovascular invasion			0.416		0.540		0.421			0.247		0.821		0.256
Absent/present	32/109	11/48		11/46		16/64		25/81	7/34		10/35		10/44	
Pathological T category			0.440		*0.003*		0.758			0.053		*0.009*		0.243
T1	16	4 (25.0)		4 (25.0)		8 (50.0)		12	1 (8.3)		4 (33.3)		3 (25.0)	
T2	23	9 (39.1)		17 (73.9)		15 (65.2)		18	7 (38.9)		14 (77.8)		10 (55.6)	
T3	34	14 (41.2)		10 (29.4)		18 (52.9)		29	12 (41.4)		9 (31.0)		14 (48.3)	
T4	68	32 (47.1)		26 (38.2)		39 (57.4)		47	21 (44.7)		18 (38.3)		27 (57.4)	
Pathological N category			0.383		1.000		0.770			0.437		0.872		0.290
N0	45	16 (35.6)		18 (40.0)		24 (53.3)		36	12 (33.3)		17 (47.2)		14 (38.9)	
N1	25	12 (48.0)		10 (40.0)		15 (60.0)		21	9 (42.9)		8 (38.1)		12 (57.1)	
N2	22	7 (31.8)		9 (40.9)		11 (50.0)		18	5 (27.8)		8 (44.4)		9 (50.0)	
N3	49	24 (49.0)		20 (40.8)		30 (61.2)		31	15 (48.4)		12 (38.7)		19 (61.3)	
Pathological TNM stage			*0.043*		0.448		0.926			*0.042*		0.356		0.160
I	26	8 (30.8)		11 (42.3)		15 (57.7)		21	5 (23.8)		11 (52.4)		8 (38.1)	
II	40	16 (40.0)		19 (47.5)		22 (55.0)		32	13 (40.6)		16 (50.0)		16 (50.0)	
III	65	27 (41.5)		23 (35.4)		38 (58.5)		48	18 (37.5)		16 (33.3)		27 (56.3)	
IV	10	8 (80.0)		4 (40.0)		5 (50.0)		5	5 (100)		2 (40.0)		3 (60.0)

We further divided 141 GC patients into three groups according to the MSI/EBV status to analyze their correlation with methylated genes: MSI+/EBV− (*n* = 12), EBV+/MSI− (*n* = 26), and MSI−/EBV− (*n* = 99). Four patients with MSI+/EBV+ were excluded because the sample size was too small for analysis. As shown in Additional file 7: Table S3 and Fig. 3a, the frequency of *ADAM19* hypermethylation was similar among the three groups, which was also observed for *MSC* hypermethylation. The frequency of *FLI1* hypermethylation was the highest in the EBV+/MSI− group, followed by the MSI+/EBV− group and MSI−/EBV− group.

#### Plasma samples

To obtain a higher sensitivity, the methylation status of the above three genes in cell-free DNA (cfDNA) was evaluated using the NGS method in 106 patients. The concentration of cfDNA extracted from plasma was 1.513 ± 3.838 ng/ul. The average methylation values of the normal control group were 0.017, 0.008, and 0.052 for the *ADAM19*, *FLI1*, and *MSC* genes, respectively. The correlations of the hypermethylation status of the three genes in plasma samples were shown in Additional file 8: Table S4 and Fig. 3b. Among these 106 patients, 41 (38.7%), 45 (42.5%), and 54 (50.9%) were found to exhibit genetic hypermethylation in cfDNA of the *ADAM19*, *FLI1*, *and MSC* genes, respectively. Patients with hypermethylation of any one of the three genes were significantly associated with hypermethylation of the other two genes (Additional file 9: Table S4). Twenty-three patients had co-hypermethylation of *ADAM19* and *FLI1*; 31 patients had co-hypermethylation of *ADAM19* and *MSC*; 31 patients had co-hypermethylation of *FLI1* and *MSC*; and 22 patients had co-hypermethylation of the three genes (Fig. 2b). As shown in Table 1, patients with *ADAM19* hypermethylation in cfDNA were more likely to have stage IV GC than those without *ADAM19* hypermethylation. Patients with *FLI1* hypermethylation in cfDNA had fewer early GCs (pT1) than those without *FLI1* hypermethylation. Patients with *MSC* hypermethylation in cfDNA had fewer superficial-type GCs than those without *MSC* hypermethylation.

The 106 patients with plasma samples were divided into three groups: MSI+/EBV− (*n* = 8), EBV+/MSI− (*n* = 21), and MSI−/EBV− (*n* = 75). Two patients with MSI+/EBV+ were excluded. Similar to the findings in patients with tissue samples, the frequency of *ADAM19* and *MSC* hypermethylation was not significantly different among the three groups. *FLI1* hypermethylation was the highest in the EBV+/MSI− group, followed by the MSI+/EBV− group and MSI−/EBV− group (Additional file 7: Table S3, Fig. 3b).

### Initial recurrence patterns

#### Tissue samples

Among the 141 patients, 119 who received curative surgery were analyzed for the initial recurrence patterns. Patients with *FLI1* hypermethylation in GC tissues were associated with more liver metastases (*P* = 0.026), more distant lymphatic metastases (*P* = 0.038), and more paraaortic lymph node metastases (*P* = 0.007) than those without *FLI1* hypermethylation. Patients with *MSC* hypermethylation in GC tissues had more liver metastases than those without *MSC* hypermethylation (*P* = 0.012). No correlation was found between the initial recurrence pattern and *ADAM19* hypermethylation in GC tissues (Table 2). Regarding the number of hypermethylated genes, patients with hypermethylation of all the three genes had significantly more liver metastases (*P* = 0.006), more distant lymphatic metastases (*P* = 0.009), and more paraaortic lymph node metastases (*P* = 0.009) than patients with hypermethylation of two or fewer of the three genes.

**Table 2:** The patterns of initial recurrence of gastric cancer after curative surgery according to the methylation status of the three genes in the tissue and plasma samples of gastric cancer patient

	Tissue samples							Plasma samples						
	Case no. *n* = 119	*ADAM19* *n* = 49	*P* value	*FLI1* *n* = 49	*P* value	*MSC* *n* = 67	*P* value	Case no. *n* = 93	*ADAM19* *n* = 36	*P* value	*FLI1* *n* = 42	*P* value	*MSC* *n* = 47	*P* value
Total recurrence	55	21 (38.2)	0.579	27 (49.1)	0.135	33 (60.0)	0.465	44	18 (40.9)	0.831	23 (52.3)	0.216	23 (52.3)	0.836
Locoregional recurrence	22	11 (50.0)	0.472	11 (50.0)	0.472	15 (68.2)	0.242	17	9 (52.9)	0.270	10 (58.8)	0.283	11 (64.7)	0.284
Hepatoduodenal ligament	10	5 (50.0)	0.739	5 (50.0)	0.739	6 (60.0)	1.000	6	3 (50.0)	0.674	4 (66.7)	0.404	3 (50.0)	1.000
Abdominal wall	13	7 (53.8)	0.378	7 (53.8)	0.378	10 (76.9)	0.144	11	6 (54.5)	0.327	7 (63.6)	0.214	9 (81.8)	0.050
Perigastric area	1	1 (100)	0.412	0	1.000	0	0.437	1	1 (100)	0.387	0	1.000	0	0.495
Anastomosis	11	4 (36.4)	1.000	7 (63.6)	0.196	7 (63.6)	0.754	10	4 (40.0)	1.000	6 (60.0)	0.339	4 (40.0)	0.523
Distant metastasis	42	16 (38.1)	0.698	20 (47.6)	0.333	27 (64.3)	0.247	32	13 (40.6)	0.825	16 (50.0)	0.519	17 (53.1)	0.828
Peritoneal dissemination	16	6 (37.5)	0.792	6 (37.5)	0.792	9 (56.3)	1.000	13	5 (38.5)	1.000	5 (38.5)	0.766	7 (53.8)	1.000
Hematogenous metastasis	20	7 (35.0)	0.624	11 (55.0)	0.215	15 (75.0)	0.084	14	6 (42.9)	0.771	8 (57.1)	0.390	7 (50.0)	1.000
Liver	12	5 (41.7)	1.000	9 (75.0)	*0.026*	11 (91.7)	*0.012*	9	4 (44.4)	0.731	7 (77.8)	*0.042*	6 (66.4)	0.486
Lung	5	1 (20.0)	0.648	0	0.077	2 (40.0)	0.652	4	1 (25.0)	1.000	0	0.124	0	0.056
Bone	6	1 (16.7)	0.399	2 (33.3)	1.000	5 (83.3)	0.230	4	1 (25.0)	1.000	1 (25.0)	0.624	2 (50.0)	1.000
Distant lymphatic recurrence	13	7 (53.8)	0.378	9 (69.2)	*0.038*	10 (76.9)	0.144	10	5 (50.0)	0.502	7 (70.0)	0.176	7 (70.0)	0.316
Virchow’s node	1	1 (100)	0.412	1 (100)	0.412	1 (100)	1.000	1	1 (100)	0.387	1 (100)	0.452	1 (100)	1.000
Lymphangitis carcinomatosa	2	1 (50.0)	1.000	0	0.511	2 (100)	0.504	2	1 (50.0)	1.000	0	0.499	1 (50.0)	1.000
Para-aortic lymph node	11	6 (54.5)	0.357	9 (81.8)	*0.007*	8 (72.7)	0.344	8	4 (50.0)	0.706	7 (87.5)	*0.021*	6 (75.0)	0.267

Some patients had more than one initial recurrence patterns

The 119 patients were divided into two groups: (MSI+ or EBV+) and MSI−/EBV−. Regarding *ADAM19* hypermethylation, there was no significant difference in the initial recurrence patterns between patients with or without *ADAM19* hypermethylation in either group. MSI−/EBV− patients with *FLI1* hypermethylation were associated with more liver metastasis than those without *FLI1* hypermethylation, while no significant difference in initial recurrence patterns was observed in MSI+ or EBV+ patients (Table 3). MSI+ or EBV+ patients with *MSC* hypermethylation were associated with more liver metastasis than those without *MSC* hypermethylation, while no significant difference in initial recurrence patterns was found in MSI−/EBV− patients (Table 3). Regarding the number of hypermethylated genes, MSI+ or EBV+ patients with at least two hypermethylated genes in the tissue samples were associated with more liver metastasis than those with less than two hypermethylated genes, which was not observed in MSI−/EBV− patients (Table 4).

**Table 3:** The initial recurrence patterns of gastric cancer patients receiving curative surgery with or without hypermethylation of *FLI1* or *MSC* in tissue samples according to MSI/EBV status

	MSI+ or EBV+ (*n* = 37)						MSI− and EBV− (*n* = 82)					
	*FLI1* hypermethylation			*MSC* hypermethylation			*FLI1* hypermethylation			*MSC* hypermethylation		
	Without *n* = 16	With *n* = 21	*P* value	Without *n* = 16	With *n* = 21	*P* value	Without *n* = 54	With *n* = 28	*P* value	Without *n* = 36	With *n* = 46	*P* value
Total recurrence	6 (37.5)	11 (52.4)	0.368	6 (37.5)	11 (52.4)	0.368	22 (40.7)	16 (57.1)	0.158	16 (44.4)	22 (47.8)	0.761
Locoregional recurrence	0	3 (14.3)	0.115	1 (6.3)	2 (9.5)	0.718	11 (20.4)	8 (28.6)	0.404	6 (16.7)	13 (28.3)	0.217
Hepatoduodenal ligament	0	1 (4.8)	0.376	1 (6.3)	0	0.245	5 (9.3)	4 (14.3)	0.490	3 (8.3)	6 (13.0)	0.498
Abdominal wall	0	2 (9.5)	0.204	0	2 (9.5)	0.204	6 (11.1)	5 (17.9)	0.395	3 (8.3)	8 (17.4)	0.232
Perigastric area	0	0	–	0	0	–	1 (1.9)	0	0.469	1 (2.8)	0	0.255
Anastomosis	1 (6.3)	4 (19.0)	0.259	2 (12.5)	3 (14.3)	0.875	3 (5.6)	3 (10.7)	0.395	2 (5.6)	4 (8.7)	0.588
Distant metastasis	5 (31.3)	8 (38.1)	0.666	3 (18.8)	10 (47.6)	0.068	17 (31.5)	12 (42.9)	0.307	12 (33.3)	17 (37.0)	0.733
Peritoneal dissemination	1 (6.3)	3 (14.3)	0.435	1 (6.3)	3 (14.3)	0.435	9 (16.7)	3 (10.7)	0.470	6 (16.7)	6 (13.0)	0.645
Hematogenous metastasis	4 (25.0)	5 (23.8)	0.933	2 (12.5)	7 (33.3)	0.143	5 (9.3)	6 (21.4)	0.125	3 (8.3)	8 (17.4)	0.232
Liver	2 (12.5)	5 (23.8)	0.384	0	7 (33.3)	*0.010*	1 (1.9)	4 (14.3)	*0.026*	1 (2.8)	4 (8.7)	0.266
Lung	2 (12.5)	0	0.096	2 (12.5)	0	0.096	3 (5.6)	0	0.204	1 (2.8)	2 (4.3)	0.707
Bone	1 (6.3)	0	0.245	0	1 (4.8)	0.376	3 (5.6)	2 (7.1)	0.776	1 (2.8)	4 (8.7)	0.266
Distant lymphatic recurrence	0	3 (14.3)	0.115	0	3 (14.3)	0.115	4 (7.4)	6 (21.4)	0.066	3 (8.3)	7 (15.2)	0.344
Virchow’s node	0	0	–	0	0	–	0	1 (3.6)	0.162	0	1 (2.2)	0.373
Lymphangitis carcinomatosa	0	0	–	0	0	–	2 (3.7)	0	0.303	0	2 (4.3)	0.205
Para-aortic lymph node	0	3 (14.3)	0.115	0	3 (14.3)	0.115	3 (3.7)	6 (21.4)	*0.010*	3 (8.3)	5 (10.9)	0.701

*MSI* microsatellite instability, *EBV* Epstein-Barr virus

**Table 4:** The initial recurrence patterns of gastric cancer patients receiving curative surgery with ≥ 2 or < 2 hypermethylated genes in tissue samples according to MSI/EBV status

	MSI+ or EBV+ (*n* = 37)			MSI− and EBV− (*n* = 82)		
	No. of hypermethylated genes			No. of hypermethylated genes		
	< 2 *n* = 31	≥ 2 *n* = 6	*P* value	< 2 *n* = 69	≥ 2 *n* = 13	*P* value
Total recurrence	13 (41.9)	4 (66.7)	0.266	33 (47.8)	5 (38.5)	0.535
Locoregional recurrence	2 (6.5)	1 (16.7)	0.401	16 (23.2)	3 (23.1)	0.993
Hepatoduodenal ligament	1 (3.2)	0	0.656	9 (13.0)	0	0.168
Abdominal wall	1 (3.2)	1 (16.7)	0.183	8 (11.6)	3 (23.1)	0.265
Perigastric area	0	0	–	1 (1.4)	0	0.662
Anastomosis	4 (12.9)	1 (16.7)	0.805	5 (7.2)	1 (7.7)	0.955
Distant metastasis	9 (29.0)	4 (66.7)	0.077	27 (39.1)	2 (15.4)	0.100
Peritoneal dissemination	2 (6.5)	2 (33.3)	0.052	12 (17.4)	0	0.104
Hematogenous metastasis	6 (19.4)	3 (50.0)	0.109	10 (14.5)	1 (7.7)	0.509
Liver	4 (12.9)	3 (50.0)	*0.034*	5 (7.2)	0	0.317
Lung	2 (6.5)	0	0.522	3 (4.3)	0	0.444
Bone	1 (3.2)	0	0.656	4 (5.8)	1 (7.7)	0.793
Distant lymphatic recurrence	2 (6.5)	1 (16.7)	0.401	9 (13.0)	1 (7.7)	0.589
Virchow’s node	0	0	–	1 (1.4)	0	0.662
Lymphangitis carcinomatosa	0	0	–	1 (1.4)	1 (7.7)	0.181
Para-aortic lymph node	2 (6.5)	1 (16.7)	0.401	8 (11.6)	0	0.196

*MSI* microsatellite instability, *EBV* Epstein-Barr virus

#### Plasma samples

Among the 106 GC patients with available plasma samples, 93 patients receiving curative surgery were enrolled for the analysis of initial recurrence patterns. Patients with *FLI1* hypermethylation in cfDNA had more liver metastases (*P* = 0.042) and more paraaortic lymph node metastases (*P* = 0.021) than those without *FLI1* hypermethylation. No correlation was found between the initial recurrence pattern and the methylation status of *ADAM19* or *MSC* in GC plasma samples (Table 2). Regarding the number of hypermethylated genes, patients with hypermethylation of all three genes had significantly more liver metastases (*P* = 0.029) and more paraaortic lymph node metastases (*P* = 0.037) than patients with hypermethylation of two or fewer of the three genes.

The 93 patients were divided into two groups according to MSI/EBV status: MSI+ or EBV+ group and MSI−/EBV− group. Regarding *ADAM19* or *MSC* hypermethylation, there was no significant difference in the initial recurrence patterns in either group of patients. MSI−/EBV− patients with *FLI1* hypermethylation in plasma samples were associated with more hematogenous metastasis and liver metastasis than those without *FLI1* hypermethylation, which was not observed in MSI+ or EBV+ patients (Table 5). Regarding the number of hypermethylated genes in plasma samples, there was no significant difference in the initial recurrence patterns in both groups.

**Table 5:** The initial recurrence patterns of gastric cancer patients receiving curative surgery with or without *FLI1* hypermethylation in plasma samples according to MSI/EBV status

	MSI+ or EBV+ (*n* = 28)			MSI− and EBV− (*n* = 65)		
	*FLI1* hypermethylation			*FLI1* hypermethylation		
	Without *n* = 10	With *n* = 18	*P* value	Without *n* = 41	With *n* = 24	*P* value
Total recurrence	6 (60.0)	9 (50.0)	0.611	15 (36.6)	14 (58.3)	0.089
Locoregional recurrence	0	3 (16.7)	0.172	7 (17.1)	7 (29.2)	0.252
Hepatoduodenal ligament	0	1 (5.6)	0.448	2 (4.9)	3 (12.5)	0.266
Abdominal wall	0	2 (11.1)	0.274	4 (9.8)	5 (20.8)	0.212
Perigastric area	0	0	–	1 (2.4)	0	0.441
Anastomosis	1 (10.0)	4 (22.2)	0.418	3 (7.3)	2 (8.3)	0.882
Distant metastasis	5 (50.0)	6 (33.3)	0.387	11 (26.8)	10 (41.7)	0.217
Peritoneal dissemination	1 (10.0)	2 (11.1)	0.927	7 (17.1)	3 (12.5)	0.622
Hematogenous metastasis	4 (40.0)	3 (16.7)	0.172	2 (4.9)	5 (20.8)	*0.045*
Liver	2 (20.0)	3 (16.7)	0.825	0	4 (16.7)	*0.007*
Lung	2 (20.0)	0	0.119	2 (4.9)	0	0.272
Bone	1 (10.0)	0	0.172	2 (4.9)	1 (4.2)	0.895
Distant lymphatic recurrence	0	2 (11.1)	0.274	3 (7.3)	5 (20.8)	0.109
Virchow’s node	0	0	–	0	1 (4.2)	0.188
Lymphangitis carcinomatosa	0	0	–	2 (4.9)	0	0.272
Para-aortic lymph node	0	2 (11.1)	0.274	1 (2.4)	5 (20.8)	*0.013*

*MSI* microsatellite instability, *EBV* Epstein-Barr virus

### Survival analysis

Among the 119 patients who received curative surgery, the 5-year OS rates did not significantly differ between patients with one or no hypermethylated genes and patients with two or three hypermethylated genes (51.8% vs. 41.7%, *P* = 0.539). Among stage III GC patients, a trend of worse 5-year OS rates was observed for patients with two or three hypermethylated genes than for those with one or no hypermethylated genes (20.0% vs. 40.0%, *P* = 0.074).

**Fig. 4:**
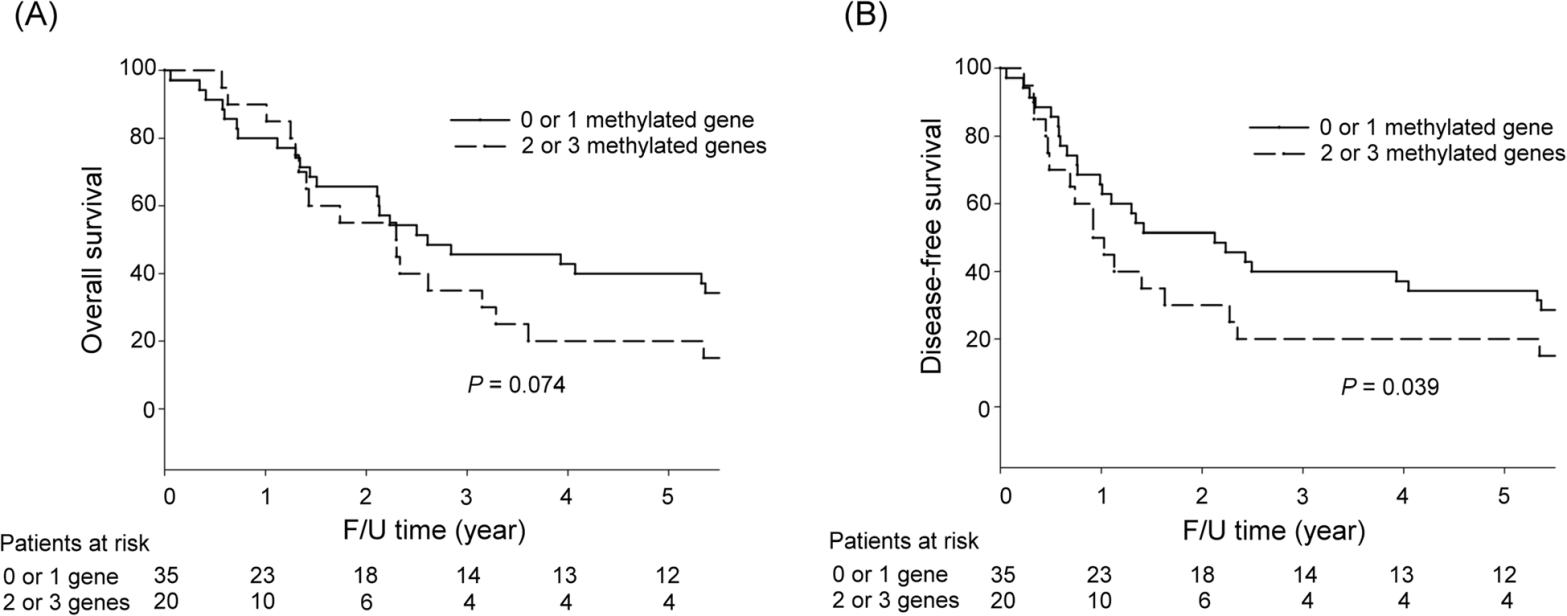
Survival curves of stage III GC patients according to the number of hypermethylated genes. **a** Five-year OS rates: a trend of a worse 5-year OS rates was observed in patients with two or three hypermethylated genes than in those with one or no hypermethylated genes. **b** Five-year DFS rates: patients with two or three hypermethylated genes had significantly worse 5-year DFS rates than those with one or no hypermethylated genes

The 5-year DFS rates did not significantly differ between patients with one or no hypermethylated genes and patients with two or three hypermethylated genes (47.0% vs. 36.1%, *P* = 0.240, Fig. 4a). In stage III GC patients, the 5-year DFS rates were significantly worse in patients with two or three hypermethylated genes than for those with one or no hypermethylated genes (20.0% vs. 34.3%, *P* = 0.039, Fig. 4b).

For subgroup analysis, MSI−/EBV− GC patients with *FLI1* hypermethylation in plasma samples had a trend of worse 5-year OS rates (33.3% vs. 56.1%, *P* = 0.104) and a significantly worse 5-year DFS rates (29.2% vs. 53.7%, *P* = 0.033) than those without *FLI1* hypermethylation, which was not observed in MSI+ or EBV+ GC. Furthermore, there was no significant difference in 5-year OS and DFS rates between patients with or without hypermethylation of *ADAM19* or *MSC* in either (MSI+ or EBV+) GC patients or MSI−/EBV− GC patients.

As shown in Additional file 9: Table S5, in both tissue and plasma samples, multivariate analysis demonstrated that *FLI1* hypermethylation, pathological TNM stage, and adjuvant chemotherapy were independent prognostic factors affecting OS. In both tissue and plasma samples, multivariate analysis showed that *FLI1* hypermethylation, pathological TNM stage, adjuvant chemotherapy, and cell differentiation were independent prognostic factors affecting DFS.

Table 6 demonstrated the summary of the prognostic value of patients with hypermethylated genes compared with those without hypermethylated genes in tissue and plasma samples.

**Table 6:** The summary of the prognostic value of patients with hypermethylated genes compared with those without hypermethylated genes in tissue and plasma samples

	*ADAM19* methylation		*FLI1* methylation		*MSC* methylation	
	Tissue	Plasma	Tissue	Plasma	Tissue	Plasma
Clinicopathological features	More stage IV GC	More stage IV GC	Fewer early GC	Fewer early GC	None	Fewer superficial GC
Initial recurrence pattern	No correlation	No correlation	More tumor recurrence in the liver, distant lymphatic and para-aortic LN	More liver metastasis	More liver metastasis	No correlation
Patient prognosis	No correlation	No correlation	Independent prognostic factor of OS and DFS	Independent prognostic factor of OS and DFS	No correlation	No correlation

*DFS* disease-free survival, *GC* gastric cancer, *LN* lymph node, *OS* overall survival

## Discussion

In the present study, we demonstrate an approach finding novel hypermethylated genes that can be used to differentiate GC from normal gastric tissue using a genome-wide high-resolution array. We then further validated these genes in tissue and plasma samples. Our novel findings showed that patients with *FLI1* hypermethylation in tissue and plasma samples were associated with more advanced GC and more liver and paraaortic lymph node metastases than those without *FLI1* hypermethylation. Only for stage III GC, patients with hypermethylation of two or three genes had a worse 5-year DFS rates than those with hypermethylation of one or none of the three genes. *FLI1* hypermethylation was an independent prognostic factor affecting OS and DFS in both tissue and plasma samples. Subgroup analysis showed that *FLI1* hypermethylation in both tissue and plasma samples was associated with liver metastasis in MSI−/EBV− GC, and *MSC* hypermethylation in tissue samples was correlated with liver metastasis in MSI+ or EBV+ GC. Patients with *FLI1* hypermethylation in plasma samples had a significantly worse 5-year DFS rate than those without *FLI1* hypermethylation in MSI−/EBV− GC.

In the present study, we used a genome-wide Illumina methylation EPIC BeadChip assay to identify novel hypermethylated genes that are differentially expressed between GC and normal gastric tissues. The three top hypermethylated genes, *ADAM19*, *FLI1*, and *MSC*, were identified and validated in tissue and plasma samples. In previous reports, *FLI1* hypermethylation in tumor tissues was observed in GC [17], colorectal adenomas, and carcinomas [7, 18]. Furthermore, *FLI1* hypermethylation was identified in 65.7% of plasma samples obtained from colorectal cancer patients [7]. The above studies indicate that *FLI1* hypermethylation could play a tumor suppressor role in both GC and colorectal cancer. Our novel findings show that *FLI1* hypermethylation is associated with fewer early GC and more liver and paraaortic lymph node metastases than is observed in those without *FLI1* hypermethylation, and these results were consistent across both tissue and plasma samples. To the best of our knowledge, most of the prognostic DNA methylation markers are hypermethylated in MSI+ GC or EBV+ GC, and the methylation status of these markers is likely to be related to the good prognosis. Despite the high incidence of positive methylation in MSI+ or EBV+ GC with good prognosis, it is unlikely that the methylation status of a specific DNA methylation marker is related to the poor prognosis of GC patients. In the subgroup analysis in the present study, although our results showed that *FLI1* hypermethylation was significantly more frequent in MSI+ or EBV+ GC than in MSI−/EBV−, *FLI1* hypermethylation in plasma was correlated with liver metastasis and a worse 5-year DFS rate in MSI−/EBV− rather than MSI+ or EBV+ GC, which was another novel finding of the present study. Consequently, *FLI1* hypermethylation was associated with GC subtypes, recurrence patterns, and poor prognosis in GC subtypes with MSI−/EBV−. Our results may remind physicians that they should be aware of the possibility of tumor recurrence and poor survival during the surveillance of specific subtypes of GC exhibiting *FLI1* hypermethylation.

*ADAM19* is a downstream target and a key component of transforming growth factor-beta (*TGF-β*) signaling. *TGF-β* can cause growth inhibition in normal ovarian surface epithelial cells, induce the nuclear translocation of *SMAD4*, and upregulate *ADMA19*. In ovarian cancer cells, the induction and nuclear translocation of *SMAD4* were negligible and refractory to *TGF-β1* stimulation, the promoter region of *ADAM19* was hypermethylated, and *ADAM19* expression was greatly reduced. These results suggest that *ADAM19* hypermethylation may contribute to ovarian cancer [19]. The novel findings in the present study demonstrated that patients with *ADAM19* hypermethylation more often exhibited stage IV GC than those without *ADAM19* hypermethylation in both tissue and plasma samples. Consequently, *ADAM19* hypermethylation indicates a high possibility of stage IV GC, which can provide an important gauge for physicians and might influence subsequent treatment modalities.

*MSC* hypermethylation was reported to be strongly associated with increasing disease severity in the histological progression from gastritis with no metaplasia, to gastritis with metaplasia, to gastric adenocarcinoma [20]. Our results showed that GC patients with *MSC* hypermethylation in plasma had, based on gross morphology, more non-superficial type tumors than were found in those without *MSC* hypermethylation. Patients with non-superficial-type gastric tumors and *MSC* hypermethylation in plasma might therefore have a high risk of GC. As a result, patients highly suspected of having GC by endoscopists but apparently lacking malignancy based on the pathology of a biopsied specimen could benefit from a test to identify *MSC* hypermethylation in plasma. In the subgroup analysis, patients with *MSC* hypermethylation were associated with more liver metastasis in MSI+ or EBV+ GC, rather than MSI−/EBV− GC, which is another novel finding of the present study. Consequently, our results might remind physicians to be aware of liver metastasis during follow-up for MSI+ or EBV+ GC exhibiting *MSC* hypermethylation.

With the combination of the three hypermethylated genes, our results demonstrated that in both tissue and plasma samples, patients with hypermethylation of all three genes had significantly more liver metastases and more paraaortic lymph node metastases than patients with hypermethylation of one, two, or none of the three genes. Moreover, especially in stage III GC, the 5-year DFS rates were significantly worse in patients with two or three hypermethylated genes than in those with one or no hypermethylated genes. Consequently, the three hypermethylated genes could serve as useful biomarkers for predicting tumor recurrence patterns and patient survival in GC patients. We hope that our methods for identifying hypermethylated markers with clinical significance can be applied for not only GC but also for other types of cancer in the future.

DNA methylation profiles at the precancerous stage may indicate tumor aggressiveness and patient survival for various cancers, including GC [21–26]. As a result, the accumulation of DNA methylation of multiple tumor-associated genes may result in carcinogenesis from the precancerous stage to cancer and even tumor progression. The investigation of DNA methylation of tumor-related genes can be helpful in early cancer detection, early detection of tumor progression, and prediction of patient prognoses. Because methylation studies are not easy to perform in the routine practice, the results of immunohistochemical staining for the three genes may be associated with the methylation status and could be used in the clinical practice, follow-up, and therapy modification for GC patients.

There are some limitations to the present study. First, this is a retrospective study, and bias could exist. Second, although our results showed that *FLI1* hypermethylation was significantly associated with recurrence patterns in GC, and stage III GC patients with two or three hypermethylated genes had worse 5-year DFS rates than those with one or no hypermethylated genes, the number of patients was limited, and bias might have occurred. In the future, we will enroll more patients, and further in vivo and in vitro studies should be performed to validate these findings. Third, we did not validate the status of the top three hypomethylated genes, including *TACSTD2*, *SIM2*, and *DAPK*, in tissue and plasma samples in the present study. Our future study will focus on the hypomethylation status of the three genes and their clinical relevance in tissue and plasma samples will be investigated as well.

## Conclusions

Our results showed that a genome-wide high-resolution array analysis may be useful for investigating novel hypermethylated genes, which can be further validated in tissue and plasma samples. DNA methylation can serve as a useful biomarker for predicting recurrence patterns and survival in both GC patients and GC subtypes. The present study demonstrated the potential clinical utility of identifying the DNA methylation status in both tissue and plasma in GCs and broader application might be achieved in other tumor types.

## Materials and methods

### Study design

**Fig. 5:**
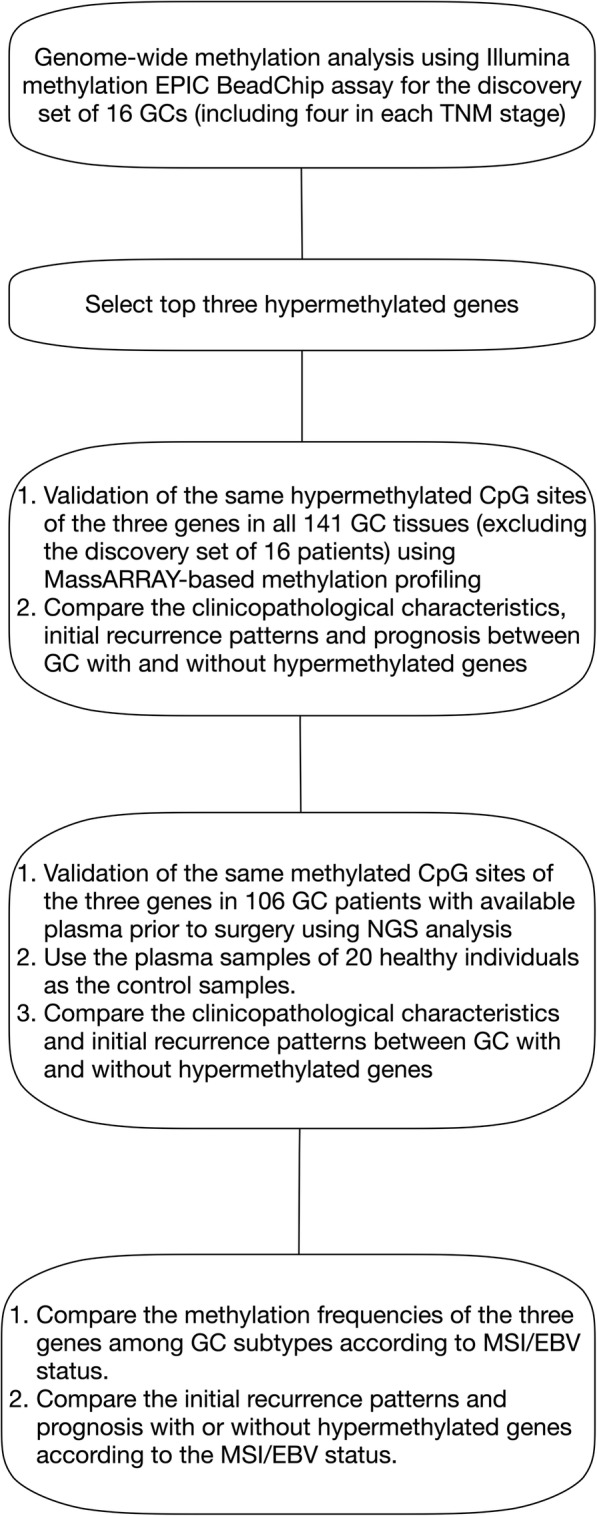
The study design

The study design is shown in Fig. 5. A total of 157 GC patients were enrolled in this study. A genome-wide methylation profiling was performed using an Illumina Methylation EPIC BeadChip assay in a discovery set of 16 GC patients, including four patients in each TNM stage. The top three hypermethylated genes at CpG sites that were differentially expressed between tumor and normal gastric tissues were selected as candidate genes. Validation of the same methylated CpG sites of the three genes as identified from the EPIC arrays was performed for tumor and normal gastric tissues in 141 GC patients (excluding the discovery set of 16 GC patients) using MassARRAY-based methylation profiling, and we further investigated the correlations between the clinicopathological characteristics and the methylation status of the three genes in all 141 patients. Validation of the same CpG sites of the three genes was additionally performed using next-generation sequencing (NGS) analysis in 106 patients for whom plasma samples prior to surgery were available. The plasma samples of 20 healthy individuals were used as the control samples. The relationships between the clinicopathological characteristics and expression profiles of the top three hypermethylated genes were investigated. Furthermore, we also performed analyses of MSI and EBV status for GC tissue samples, and GC patients were divided into subtypes according to MSI/EBV status. The methylation frequencies of the three genes and the initial recurrence patterns were compared among the GC subtypes.

### Patient selection and surgical treatment

Between April 2005 and December 2011, a total of 157 GC patients who underwent surgery for GC were enrolled in this study. Informed written consent was obtained from all enrolled patients, and this study was approved by the Institutional Review Board at our institute.

Pathological staging of GC was performed according to the eighth American Joint Committee on Cancer/Union for International Cancer Control (AJCC/UICC) TNM classification [27]. All operations were performed by surgeons who specialized in GC. The data were prospectively collected and recorded using a computer, and the patients’ follow-up conditions were regularly updated.

### Follow-up

Overall survival was calculated from the time of surgery until death or the date of the last follow-up. None of the patients received preoperative chemotherapy. Before 2008, neither adjuvant chemotherapy nor radiotherapy was routinely performed after curative surgery; rather, these procedures were performed only when tumor recurrence was diagnosed or highly suspected. Since 2008, adjuvant therapy (such as TS-1) has been prescribed for stage II or stage III patients who underwent curative surgery in our hospital. Among the 141 patients (validation set), 119 patients received curative surgery, including 95 patients who were stage II or stage III. Sixteen out of the 95 patients received postoperative adjuvant chemotherapy, including TS-1 for one patient and 5-FU-based intravenous chemotherapy for 15 patients. Among the total 141 patients enrolled in the present study, two patients received surgery after 2008, including one stage II and one stage III GC; the stage III GC patient was the only one patient receiving TS-1 therapy.

Follow-up assessments were performed every 3 months for the first 5 years after surgery and every 6 months thereafter until the patient’s death. The follow-up procedures included medical histories, physical examinations, routine blood tests, liver function tests, measurements of tumor marker levels (carcinoembryonic antigen and carbohydrate antigen 19-9), chest radiography, abdominal sonography, or CT scan.

### DNA extraction

DNA was extracted from tissue specimens and 800 ul of plasma using the QIAamp DNA Tissue Kit and MinElute Virus Kit (Qiagen, Valencia, CA, USA), respectively, according to the manufacturer’s recommendations. For plasma isolation, whole blood was centrifuged at 2600 rpm for 10 min at 10 °C. The collected material was centrifuged once again in 2-ml low-bind tubes at 14,500 rpm for 10 min to remove residual cells. All samples were stored at − 20 °C until cfDNA isolation. DNA quality and quantity were confirmed using a NanoDrop 1000 Spectrophotometer (Thermo Scientific) and Qubit Fluorometer (Thermo Scientific), respectively.

### MSI analysis

As mentioned in a previous study [28], the DNA of normal and tumor tissues was extracted, purified, and then amplified using a fluorescent PCR. Five reference microsatellite markers (D5S345, D2S123, D17S250, BAT25, and BAT26) were used for the analysis of microsatellite instability (MSI). MSI+ was defined when samples had ≥ 2 loci of instability with five markers. MSI− was defined as samples with one MSI or without MSI.

### EBV DNA detection based on MassARRAY

As reported in a previous study [29], EBV DNA assays were carried out using the MassARRAY system (Agenda, San Diego, CA, USA). The PCR and single-base extension primers were designed using the MassARRAY Assay Design 3.1 software, and one multiplex reaction was designed to detect the EBV virus DNA segment.

### Genome-wide methylation analysis

A total of 600 ng of genomic DNA was obtained from the tumors and adjacent non-tumor tissues of 16 GC patients (four in each TNM stage) and subjected to epigenotyping using a genome-wide Illumina methylation EPIC BeadChip kit according to the manufacturer’s instructions. After bisulfate conversion, two criteria were required in the subsequent Illumina methylation beadarray: ssDNA concentration > 10 ng/μl using a Quant-iT ssDNA assay kit (Thermo Fisher) and positive PCR results for cg05524038 or cg27640254. Then, whole-genome amplification, enzymatic fragmentation, precipitation, resuspension, and hybridization were performed, and the intensity data were acquired using an Illumina HiScan scanner. The resulting image was processed using the GenomeStudio Methylation module (Illumina) to obtain the β value for each CpG site [4]. Hypomethylation was defined as a DiffScore ≤ − 30 and a delta β value < − 0.2, while hypermethylation was defined as a DiffScore ≥ 30 and a delta β value > 0.2 [4, 30].

### MassARRAY-based methylation profiling

The top three hypermethylated genes were *ADAM19*, *FLI1*, and *MSC*, and methylation profiling was performed in these three genes using a MassARRAY EpiTYPER (Agena Bioscience, San Diego, CA, USA) based on MassCLEAVE base-specific cleavage and matrix-assisted laser desorption ionization-time of flight (MALDI-TOF) mass spectrometry. Primers were designed using the Epidesigner online software, and the quantitative methylation data for each CpG site or aggregates of multiple CpG sites were obtained from MassARRAY and analyzed using EpiTYPER software (Agena Bioscience, San Diego, CA, USA). MassARRAY and NGS were used to quantify methylation states. Tumor and adjacent normal tissues were both used to perform methylation profiling, and the average methylation value of CpG sites was used to measure methylation. If the methylation value was more than the mean + 2SD of the normal control group, the case was defined as hypermethylation.

### NGS-based target gene methylation sequencing

To detect the methylation status of candidate CpG sites of interest in cell-free DNA (cfDNA) extracted from plasma samples prior to surgery, amplicon-based enrichments for Illumina NGS sequencers were used to obtain a higher sensitivity. EpiTect Control DNA (Qiagen) was used to evaluate the performance of the target methylation sequencing design. The values of all target methylation sites were 0.98 ± 0.05 and 0.01 ± 0.02 in methylated and unmethylated controls, respectively, indicating the quantification abilities of the primer and read data were acceptable for this study. Bisulfite-converted cfDNA (10 ng) was obtained from each individual and used to amplify target CpG regions (amplicon sizes, 80–120 bp). Then, the amplicons of each sample were pooled after Ampure XP bead-based PCR purification (Beckman) and Qubit-based quantification (Thermo Fisher). The pooled amplicons were used to construct a sample library with a Roche KAPA Library Preparation Kit (Roche). The amplified libraries were quantified using a qPCR system and pooled into a single 1.5-ml tube to obtain a 10-nM pooled DNA library. The final pool was used for sequencing (Illumina MiniSeq sequencer, 2 × 150 bp). The raw output for each individual patient was 20 Mb, and the average depth of the target regions was > 1000×. In addition to cancer patients, the cfDNA of 20 healthy individuals (normal control group) was also used to perform target methylation sequencing using the above method. Finally, we directly evaluated the methylation status by analyzing read sequence-identified variants, and CpG sites for which the average methylation percentage of the target was larger than the mean + 2SD of that in the control group were considered hypermethylated phenotypes and included in the statistical analysis.

### Statistical analysis

Statistical analyses were performed using IBM SPSS version 24.0. Categorical data were compared using a *χ*^2^ test with Yates’ correction or Fisher’s exact test. The overall survival (OS) was measured from the operation date to the date of death or the final follow-up. Disease-free survival (DFS) was defined as the length of time after GC surgery during which a patient survived without tumor recurrence. The distributions of OS and DFS were estimated using the Kaplan-Meier method. Multivariate analysis using Cox proportional hazards models was performed to explore the association of the clinical parameters with OS and DFS. A *P* value of < 0.05 was considered to be statistically significant.

## Supplementary information

**Additional file 1:** The raw data of the results of genome-wide methylation profiling using an Illumina Methylation EPIC BeadChip assay.

**Additional file 2:** The data of the hypermethylated and hypomethylated genes that were differentially expressed between the normal and tumor tissues in the discovery set of 16 GC patients.

**Additional file 3:** The raw data of the clinical profile of the discovery set of 16 GC patients.

**Additional file 4:** The raw data of the clinical profile of the validation set of 141 GC patients.

**Additional file 5: Table S1.** Clinical profile in the discovery set and validation set of gastric cancer patients.

**Additional file 6: Table S2.** The frequency of methylation of the three genes in tissue and plasma samples according to the status of MSI/EBV.

**Additional file 7: Table S3.** The correlations between the three hypermethylated genes in tissue samples.

**Additional file 8: Table S4.** The correlations between the three hypermethylated genes in plasma samples.

**Additional file 9: Table S5.** Multivariate analysis of factors affecting OS and DFS of GC patients after curative surgery in tissue and plasma samples.

## Data Availability

The datasets generated and/or analyzed during the current study are available for non-commercial use from the corresponding author on reasonable request.

## Abbreviations

ADAM19: A Disintegrin and Metalloproteinase 19
AJCC: American Joint Committee on Cancer
CT: Computed tomography
DFS: Disease-free survival
EBV: Epstein-Barr virus
FLI1: Friend Leukemia Integration 1
GC: Gastric cancer
HP: Helicobacter pylori
MALDI-TOF: Matrix-assisted laser desorption ionization-time of flight
MSC: Musculin
MSI: Microsatellite instability
MSI+: Microsatellite instability-high
NGS: Next-generation sequencing
OS: Overall survival
PCR: Polymerase chain reaction
TCGA: The Cancer Genome Atlas
TGF-β: Transforming growth factor-beta
UICC: Union for International Cancer Control
