# Supplementary material for: Analysis of the clinical significance of DNA methylation in gastric cancer based on a genome-wide high-resolution array
Source: Clin Epigenetics. 2019 Nov 1;11:154. doi: 10.1186/s13148-019-0747-5 (PMC6824057; doi:10.1186/s13148-019-0747-5)
Supplement: Supplementary file 5 — Additional file 5: Table S1. Clinical profile in the discovery set and validation set of gastric cancer patients. [file 13148_2019_747_MOESM5_ESM.docx]

Table S1. Clinical profile in the discovery set and validation set of gastric cancer patients.

| Variables | Discovery set  n=16 | Validation set  n=141 | *P* value |
| --- | --- | --- | --- |
| Age (years) |  |  | 0.375 |
| <65/≧65 | 4/12 | 51/90 |  |
| Gender |  |  | 0.531 |
| Male/Female | 11/5 | 107/34 |  |
| Tumor size (cm) |  |  | 0.121 |
| <5/≧5 | 7/9 | 36/105 |  |
| Cell differentiation |  |  | 0.821 |
| Poor/moderate/well | 7/9/0 | 71/69/1 |  |
| Gross appearance |  |  | 0.654 |
| Superficial type | 2 (12.5) | 18 (12.8) |  |
| Borrmann type 1&2 | 5 (31.3) | 30 (21.3) |  |
| Borrmann type 3&4 | 9 (56.3) | 93 (66.0) |  |
| Lauren’s classification |  |  | 0.738 |
| Intestinal/diffuse type | 10/6 | 94/47 |  |
| Lymphovascular invasion | 11 (68.8) | 109 (77.3) | 0.445 |
| Pathological T category |  |  | 0.215 |
| T1 | 3 (18.8) | 16 (11.3) |  |
| T2 | 1 (6.3) | 23 (16.3) |  |
| T3 | 9 (56.3) | 34 (24.1) |  |
| T4 | 3 (18.8) | 68 (48.2) |  |
| Pathological N category |  |  | 0.922 |
| N0 | 6 (37.5) | 45 (31.9) |  |
| N1 | 2 (12.5) | 25 (17.7) |  |
| N2 | 2 (12.5) | 22 (15.6) |  |
| N3 | 6 (37.5) | 49 (34.8) |  |
| Pathological TNM Stage |  |  | 0.072 |
| I | 4 (25.0) | 26 (18.4) |  |
| II | 4 (25.0) | 40 (28.4) |  |
| III | 4 (25.0) | 65 (46.1) |  |
| IV | 4 (25.0) | 10 (7.1) |  |
| *ADAM19* hypermethylation | 14 (87.5) | 59 (41.8) | **0.001** |
| *FLI1* hypermethylation | 11 (68.8) | 57 (40.4) | **0.030** |
| *MSC* hypermethylation | 13 (81.3) | 80 (56.7) | 0.059 |
