# Supplementary material for: Analysis of the clinical significance of DNA methylation in gastric cancer based on a genome-wide high-resolution array
Source: Clin Epigenetics. 2019 Nov 1;11:154. doi: 10.1186/s13148-019-0747-5 (PMC6824057; doi:10.1186/s13148-019-0747-5)
Supplement: Supplementary file 6 — Additional file 6: Table S2. The frequency of methylation of the three genes in tissue and plasma samples according to the status of MSI/EBV. [file 13148_2019_747_MOESM6_ESM.docx]

Table S2. The correlations between the three hypermethylated genes in tissue samples.

|  |  | *ADAM19* hypermethylation | | |  | *FLI1* hypermethylation | | |
| --- | --- | --- | --- | --- | --- | --- | --- | --- |
|  |  | - | + | *P* value |  | - | + | *P* value |
| *FLI1* hypermethylation |  |  |  | **0.013** |  | - |  |  |
| - |  | 56 (68.3) | 28 (47.5) |  |  |  |  |  |
| + |  | 26 (31.7) | 31 (52.5) |  |  |  |  |  |
| *MSC* hypermethylation |  |  |  | 0.057 |  |  |  | **<0.001** |
| - |  | 41 (50.0) | 20 (33.9) |  |  | 47 (56.0) | 14 (24.6) |  |
| + |  | 41 (50.0) | 39 (66.1) |  |  | 37 (44.0) | 43 (75.4) |  |
