# Supplementary material for: Analysis of the clinical significance of DNA methylation in gastric cancer based on a genome-wide high-resolution array
Source: Clin Epigenetics. 2019 Nov 1;11:154. doi: 10.1186/s13148-019-0747-5 (PMC6824057; doi:10.1186/s13148-019-0747-5)
Supplement: Supplementary file 7 — Additional file 7: Table S3. The correlations between the three hypermethylated genes in tissue samples. [file 13148_2019_747_MOESM7_ESM.docx]

Table S3. The frequency of methylation of the three genes in tissue and plasma samples according to the status of MSI/EBV

| Genetic hypermethylation |  | Tissue samples  n=137 | | | |  | Plasma samples  n=104 | | | |
| --- | --- | --- | --- | --- | --- | --- | --- | --- | --- | --- |
|  |  | MSI/EBV  +/-  n=12 | EBV/MSI  +/-  n=26 | MSI/EBV  -/-  n=99 | *P* value |  | MSI/EBV  +/-  n=8 | EBV/MSI  +/-  n=21 | MSI/EBV  -/-  n=75 | *P* value |
| *ADAM19* hypermethylation |  | 5 (41.7) | 12 (46.2) | 41 (41.4) | 0.909 |  | 3 (37.5) | 8 (38.1) | 30 (40.0) | 0.981 |
| *FLI1* hypermethylation |  | 5 (41.7) | 17 (65.4) | 33 (33.3) | **0.012** |  | 4 (50.0) | 15 (71.4) | 26 (34.7) | **0.010** |
| *MSC* hypermethylation |  | 7 (58.3) | 14 (53.8) | 56 (56.6) | 0.958 |  | 4 (50.0) | 9 (42.9) | 40 (53.3) | 0.696 |

MSI: microsatellite instability; EBV: Epstein-Barr virus
