# Supplementary material for: Analysis of the clinical significance of DNA methylation in gastric cancer based on a genome-wide high-resolution array
Source: Clin Epigenetics. 2019 Nov 1;11:154. doi: 10.1186/s13148-019-0747-5 (PMC6824057; doi:10.1186/s13148-019-0747-5)
Supplement: Supplementary file 8 — Additional file 8: Table S4. The correlations between the three hypermethylated genes in plasma samples. [file 13148_2019_747_MOESM8_ESM.docx]

Table S4. The correlations between the three hypermethylated genes in plasma samples.

|  |  | *ADAM19* hypermethylation | | |  | *FLI1* hypermethylation | | |  |
| --- | --- | --- | --- | --- | --- | --- | --- | --- | --- |
|  |  | - | + | *P* value |  | - | + | *P* value |  |
| *FLI1* hypermethylation |  |  |  | **0.024** |  |  |  |  |  |
| - |  | 43 (66.2) | 18 (43.9) |  |  |  |  |  |  |
| + |  | 22 (33.8) | 23 (56.1) |  |  |  |  |  |  |
| *MSC* hypermethylation |  |  |  | **<0.001** |  |  |  | **0.002** |  |
| - |  | 42 (64.6) | 10 (24.4) |  |  | 38 (62.3) | 14 (31.3) |  |  |
| + |  | 23 (35.4) | 31 (75.6) |  |  | 23 (37.7) | 31 (68.9) |  |  |
