# Supplementary material for: Analysis of the clinical significance of DNA methylation in gastric cancer based on a genome-wide high-resolution array
Source: Clin Epigenetics. 2019 Nov 1;11:154. doi: 10.1186/s13148-019-0747-5 (PMC6824057; doi:10.1186/s13148-019-0747-5)
Supplement: Supplementary file 9 — Additional file 9: Table S5. Multivariate analysis of factors affecting OS and DFS of GC patients after curative surgery in tissue and plasma samples. [file 13148_2019_747_MOESM9_ESM.docx]

Table S5. Multivariate analysis of factors affecting OS and DFS of GC patients after curative surgery in tissue and plasma samples

|  | Tissue | | | | | | |  | Plasma | | | | | | |
| --- | --- | --- | --- | --- | --- | --- | --- | --- | --- | --- | --- | --- | --- | --- | --- |
|  | OS | | |  | DFS | | |  | OS | | |  | DFS | | |
|  | HR | 95% CI | *P* value |  | HR | 95% CI | *P*  value |  | HR | 95% CI | *P* value |  | HR | 95% CI | *P* value |
| Age (y/o) | 0.95 | 0.591-1.526 | 0.830 |  | 0.73 | 0.414-1.282 | 0.272 |  | 1.22 | 0.699-2.137 | 0.916 |  | 1.11 | 0.647-1.899 | 0.708 |
| Gender | 0.80 | 0.454-1.409 | 0.440 |  | 0.90 | 0.566-1.422 | 0.644 |  | 1.04 | 0.522-2.064 | 0.482 |  | 0.95 | 0.481-1.856 | 0.869 |
| Tumor size (cm) | 1.30 | 0.755-2.230 | 0.345 |  | 1.32 | 0.778-2.224 | 0.306 |  | 1.15 | 0.636-2.068 | 0.649 |  | 1.23 | 0.696-2.183 | 0.474 |
| Tumor location | 1.14 | 0.868-1.483 | 0.355 |  | 1.12 | 0.858-1.461 | 0.405 |  | 1.15 | 0.851-1.552 | 0.366 |  | 1.09 | 0.814-1.472 | 0.551 |
| Cell differentiation | 0.74 | 0.466-1.177 | 0.204 |  | 0.60 | 0.375-0.955 | **0.031** |  | 0.65 | 0.378-1.126 | 0.125 |  | 0.48 | 0.275-0.852 | **0.012** |
| Lymphovascular invasion | 1.05 | 0.539-2.052 | 0.882 |  | 0.70 | 0.355-1.366 | 0.292 |  | 0.88 | 0.413-1.882 | 0.744 |  | 0.50 | 0.231-1.099 | 0.085 |
| Pathological TNM stage | 1.84 | 1.229-2.739 | **0.003** |  | 1.93 | 1.273-2.912 | **0.002** |  | 2.01 | 1.258-3.222 | **0.004** |  | 2.05 | 1.258-3.330 | **0.004** |
| Adjuvant chemotherapy | 2.71 | 1.400-5.250 | **0.003** |  | 4.60 | 2.333-9.081 | **<0.001** |  | 4.47 | 2.053-9.732 | **<0.001** |  | 6.78 | 3.070-14.970 | **<0.001** |
| *ADAM19* hypermethylation | 0.78 | 0.488-1.234 | 0.284 |  | 0.75 | 0.470-1.190 | 0.220 |  | 1.11 | 0.632-1.931 | 0.726 |  | 1.04 | 0.591-1.821 | 0.899 |
| *FLI1* hypermethylation | 1.72 | 1.052-2.806 | **0.030** |  | 2.13 | 1.306-3.464 | **0.002** |  | 2.28 | 1.263-4.114 | **0.006** |  | 2.78 | 1.542-5.005 | **0.001** |
| *MSC* hypermethylation | 1.15 | 0.724-1.818 | 0.558 |  | 1.33 | 0.850-2.078 | 0.213 |  | 0.82 | 0.462-1.450 | 0.492 |  | 1.00 | 0.568-1.769 | 0.993 |

OS: overall survival; DFS: disease-free survival; HR: hazard ratio; CI: confidence interval
